# 2-Methoxy-1,4-naphthoquinone (MNQ) regulates cancer key genes of MAPK, PI3K, and NF-κB pathways in Raji cells

**DOI:** 10.5808/gi.21041

**Published:** 2022-03-31

**Authors:** Teck Yew Wong, Subramaniam Menaga, Chi-Ying F. Huang, Siong Hock Anthony Ho, Seng Chiew Gan, Yang Mooi Lim

**Affiliations:** 1 Centre for Cancer Research, Universiti Tunku Abdul Rahman, 43000 Kajang, Selangor, Malaysia; 2 Institute of Biopharmaceutical Sciences, National Yang-Ming University, Taipei 112, Taiwan, R.O.C.; 3 School of Biosciences, Taylor’s University, Lakeside Campus 1, 47500 Subang Jaya, Malaysia; 4 Department of Pre-Clinical Sciences Faculty of Medicine and Health Sciences, Universiti Tunku Abdul Rahman, 43000 Kajang, Malaysia

**Keywords:** 2-methoxy-1,4-naphthoquinone, cancer chemoprevention, MAPK pathway, nuclear factor-kappa B pathway, PI3K pathway

## Abstract

2-Methoxy-1,4-naphthoquinone (MNQ) has been shown to cause cytotoxic towards various cancer cell lines. This study is designed to investigate the regulatory effect of MNQ on the key cancer genes in mitogen-activated protein kinase, phosphoinositide 3-kinase, and nuclear factor кB signaling pathways. The expression levels of the genes were compared at different time point using polymerase chain reaction arrays and Ingenuity Pathway Analysis was performed to identify gene networks that are most significant to key cancer genes. A total of 43 differentially expressed genes were identified with 21 up-regulated and 22 down-regulated genes. Up-regulated genes were involved in apoptosis, cell cycle and act as tumor suppressor while down-regulated genes were involved in anti-apoptosis, angiogenesis, cell cycle and act as transcription factor as well as proto-oncogenes. MNQ exhibited multiple regulatory effects on the cancer key genes that targeting at cell proliferation, cell differentiation, cell transformation, apoptosis, reduce inflammatory responses, inhibits angiogenesis and metastasis.

## Introduction

The process of carcinogenesis is relay on over-expression of cancer genes through gene amplification, inappropriate expression of normal genes, or mutations in genes [1,2]. Accumulation of genetic mutations and epigenetic alterations are needed to induce a normal cell to a transformed cell [3] and eventually lead to cancer formation. Cancer is mainly about the active and “gain-of-function” process of the oncogenes with loss of function of tumor suppressor genes [4]. The acquisition of oncogenes and tumor suppressor genes was shown at different times during the tumor progression in different type of tumors [5].

The complication of carcinogenesis involves several cancer hallmarks. These underlying principles are describing how normal cells transform to malignant or tumor cells. Hallmarks of cancer refers to behaviors of cancer cells that sustained to proliferative signals, resisted to cell death signals, evading from growth suppressors, enabling replicative immortality, genome instability and mutation, continues tumor-promoting inflammation, deregulating cellular energetic, avoiding immune destruction, inducing angiogenesis, and activating invasion and metastasis [5]. Multiple signaling pathways regulate each aforementioned cancer hallmark.

Cancer cells are known to have alterations in multiple cellular signaling pathways involving the affected genes [6]. Aberrant cancer signaling pathways always have been characterized with their complexities regarding the alterations and deregulations of the genes involved [7]. These multiple dysfunctional signaling pathways implicated in cancer development have become one of the factors that lead to the complexity of carcinogenesis and difficulties faced in cancer research. With the understanding of cancer complexities in the link between these signaling pathways, the search for curing cancer can possibly done by disrupting these altered signaling pathways [8]. Non-toxic natural products can be used to disrupt these altered signaling pathways; these natural products can activate cell death signals in pre-cancerous or cancerous cells without affecting the normal cells [9]. Besides, these natural products can be used alone or in combination with chemotherapeutic drugs.

More than 10 cancer signaling pathways have been identified [10]. Studies on the aberrant signaling pathways are not necessarily limited to only one or two specific signaling pathways towards cancer progression. In many cases, a few of the pathways would be studied altogether to clearly know the effects of the complexities and the interactions between these signaling networks towards carcinogenesis. In term of inhibiting signal transduction, suppression of multiple signaling pathways is better than suppression on single pathway at a time [11]. In a feedback loop theory, suppression of one signaling pathway would activate another pathway automatically. This is a phenomenon where cancer cells prolong the survival as mutated cell [12]. In all, better understanding and accurate dissection of all these signaling pathways are important; this is because they are vital for elucidating the most appropriate and reliable target molecules for the design of effective cancer therapy [13]. For instance, many potential chemo preventive drugs have been tested on multiple dysfunctional proteins that are involved in multiple dysfunctional cancer signaling pathways.

*Impatiens balsamina*, Linn from the family of Balsaminaceae, is an ornamental plant that has been used to treat various skin diseases locally. 2-Methoxy-1,4-naphthoquinone (MNQ), isolated from pericarps of *Impatiens balsamina* Linn. has been shown to cause cytotoxic towards various cancer cell lines [14], show anti-tumor-promoting activity in HepG2 and Raji cells [15,16], trigger apoptotic pathway and the upper stream modulator of many cancer pathways [17], inhibit protein kinase C expressions in Raji cells [16], and suppress the invasion and migration of MDA-MB-231 [18]. Furthermore, MNQ also has been shown to inhibit WNT signaling in STF/293 cells [19], altered proteins related to cytoskeletal functions and regulations, mRNA processing, protein modifications, and oxidative stress responses [20].

PKC is one of the protein kinases that play a role in carcinogenesis and maintenance of malignant phenotype. Moreover, it is also the core upper stream kinase involved in mitogen-activated protein kinase (MAPK), phosphoinositide 3-kinase (PI3K), and nuclear factor кB (NF-κB) pathways that regulate many down-stream proteins implicated in these cancer signaling pathways. In our previous study, MNQ has been demonstrated to inhibit PKC βI, δ, and ζ [16]; therefore, this study was continued to reveal the genes that are possibly regulated by MNQ in MAPK, PI3K, and NF-κB pathways.

## Methods

### Chemicals

Phorbol 12-myristate 13-acetate (PMA) and sodium *n*-butyrate (S*n*B) were obtained from Sigma. MNQ was isolated from the pericarps of *Impatiens balsamina*, Linn. The compound was prepared in dimethyl sulfoxide and stored at 4ºC. The reagents obtained from Qiagen (Hilden, Germany) were RT^2^ First Strand Kit (containing GE buffer, 5× BC3 buffer, RE3 reverse transcriptase mix, P2 control, and RNase-free water), RT^2^ STBR Green ROX FAST mastermix (containing HotStart DNA Taq Polymerase, PCR buffer, dNTP mix [dATP, dCTP, dGTP, dTTP], SYBR Green dye ROX mastermix).

### Cell culture

Raji cells obtained from Riken Cell Bank, Japan were maintained in commercial Roswell Park Memorial Institute Media (RPMI-1640) supplemented with 10% fetal bovine serum (FBS) (Thermo Fisher, Waltham, MA, USA). The cells were incubated in a humidified atmosphere at 37°C with 5% CO_2_ incubator (ESCO, Hatboro, PA, USA).

### Structure confirmation of MNQ

The high-performance liquid chromatography (HPLC) chromatogram of the isolated MNQ was first identified by comparing the HPLC chromatogram of MNQ HPLC chromatogram obtained by Teng [14] in our previous work. After that, mass spectrometry spectrum was compared to NIST mass spectral data. The identified MNQ was then isolated and subjected to HPLC spiking analysis and gas chromatography and mass spectrometry spiking analysis. Isolated MNQ structure was confirmed with nuclear magnetic resonance (NMR). Proton and carbon NMR chemical shift results were compared to Teng [14].

### Treatment of cells for RT^2^ Profiler PCR arrays

Raji cells (5 × 10^5^ cells/mL) were incubated in 1 mL of RPMI 1640 medium (supplemented with 10 % FBS) containing 0.05 µM PMA, 3 mM SnB, and MNQ in a 24-well plate and then incubated at 37°C for 6, 12, 24, and 48 h in a CO_2_ incubator. PMA and SnB was used as inducer and enhancer respectively to cause Epstein-Barr virus activation in Raji cells and directly transform Raji cells into promotion stage.

### RNA extraction and cDNA preparation

RNA of the treated Raji cells was extracted according to the manufacturer’s instruction using RNeasy Mini Kit (Qiagen). RT^2^ RNA QC PCR Array was used to confirm the purity of RNA and excluded substandard samples prior to RT^2^ Profiler PCR Arrays analysis. The RNA samples extracted were at high-quality RNA, in which the A260:A230 ratio obtained was greater than 2.0 and the A260:A280 ratio attained was in between 1.8 to 2.0. Next, the cDNA was synthesized using the RT^2^ First Strand Kit (Qiagen) following the manufacturer’s instructions. The genomic DNA elimination mix was prepared accordingly to the manual given by Qiagen.

### RT^2^ Profiler PCR Array

The cDNA was added with RT^2^ SYBR Green ROX FAST Mastermix according to manufacturer’s protocol. RT^2^ Profiler PCR Array for the detection of key cancer genes regulation in MAPK, PI3K, and NF-κB pathways were tested in three independent experiments. The genes expression results were calculated using results generated by the RT cycler and the ∆∆C_T_ method. The C_T_ values for all wells were exported to PCR Array Data Analysis Template Excel sheet (downloaded at http://www.sabiosciences.com/dataanalysis.php) and uploaded to web-based software at www.sabiosciences.com/pcrarraydataanalysis.php. This software helps in calculating C_T_ values collected and shows p-values and fold change for each gene. The connection between genes was analyzed using web-based resources Gene Network Central Pro at http://gncpro.sabiosciences.com/gncpro/gncpro.php.

### Pathway and network analysis

The identified key cancer genes were first uploaded onto Qiagen’s Ingenuity Pathway Analysis (IPA) system for core analysis and then overlaid with the global molecular network in the ingenuity pathway knowledge base. IPA was performed to identify canonical pathways, diseases and functions, and gene networks that are most significant to key cancer genes and to categorize differentially expressed genes in specific molecular and cellular functions. Thresholds of two-fold or greater in changes in expression and a p-value of 0.05 or less for significance were used to filter the findings from the analysis with IPA software.

### Statistical analysis

The results were obtained from three separate experiments. In terms of the sensitivity and accuracy, the cutoff fold change (2^−ΔΔCt^) was set at greater than 5.0 or less than −4.0.

## Results

### Effect of MNQ on MAPK, PI3K, and NF-κB pathway-focused gene expression profiling

**Fig. 1.**
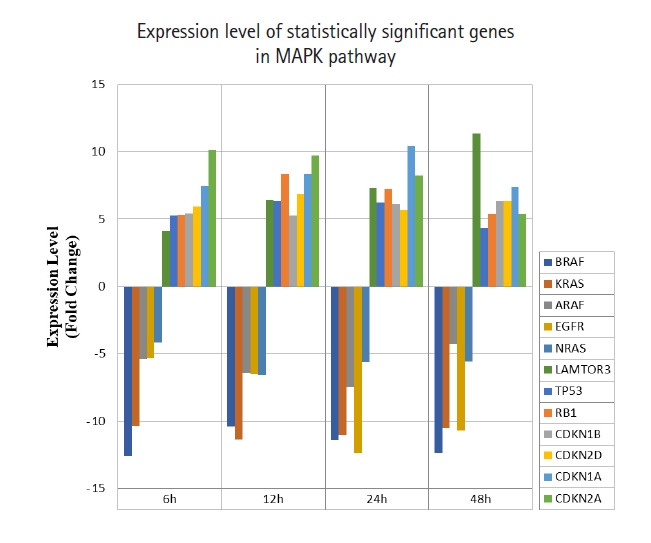
Fold change of statistically significant genes regulated by MNQ in MAPK pathway. All experiments were performed individually with triplicate. p < 0.05 and fold change > 4.0 was set as the significant threshold. MNQ, 2-methoxy-1,4-naphthoquinone; MAPK, mitogen-activated protein kinase.

**Fig. 2.**
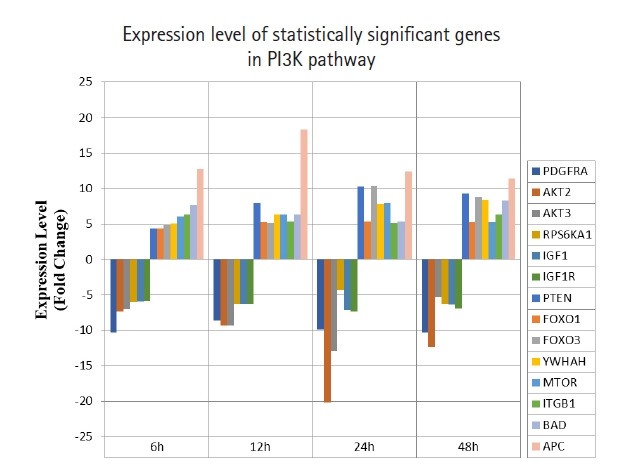
Fold change of statistically significant genes regulated by MNQ in PI3K pathway. All experiments were performed individually with triplicate. p < 0.05 and fold change > 4.0 was set as the significant threshold. MNQ, 2-methoxy-1,4-naphthoquinone; PI3K, phosphoinositide 3-kinase.

**Fig. 3.**
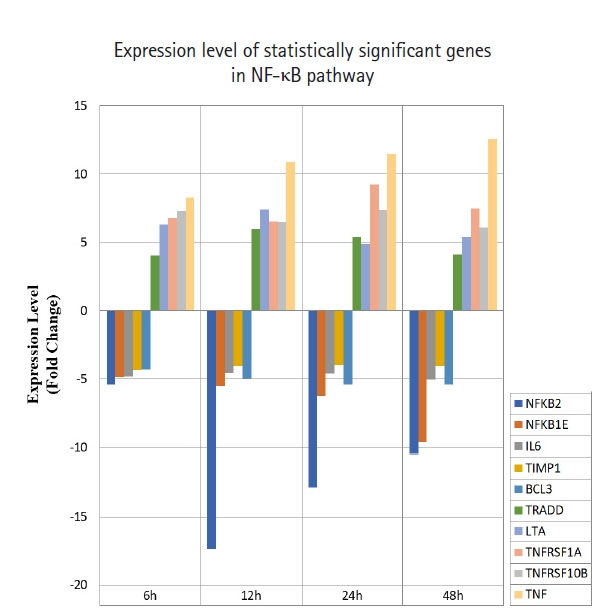
Fold change of statistically significant genes regulated by MNQ in NF-κB pathway. All experiments were performed individually with triplicate. p < 0.05 and fold change > 4.0 was set as the significant threshold. MNQ, 2-methoxy-1,4-naphthoquinone; NF-κB, nuclear factor кB.

**Fig. 4.**
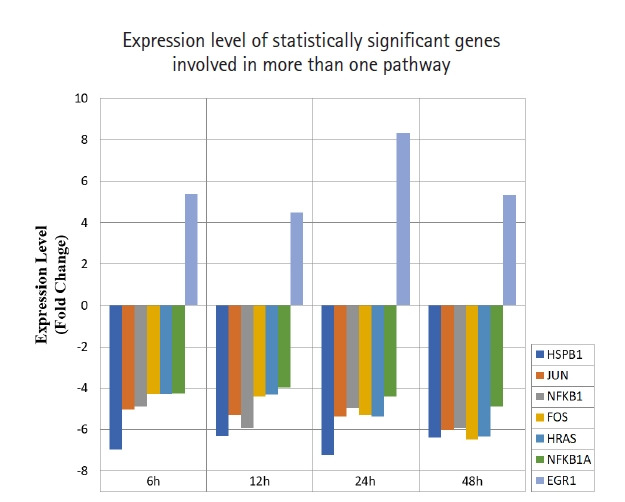
Fold change of statistically significant genes regulated by MNQ. These genes are involved in more than a pathway. All experiments were performed individually with triplicate. p < 0.05 and fold change > 4.0 was set as the significant threshold. MNQ, 2-methoxy-1,4-naphthoquinone.

A total of 43 statistically significant expressed genes were identified, which was 19.72 % of the total of 218 cancer key genes studied. All the 43 genes were statistically significant expressed in Raji cells after treated with 0.05 μM PMA, 3 mM SnB, and 40 μM MNQ (p < 0.05, fold change > 4.0). The fold changes of all statistically significant genes involved in these pathways are shown in Fig. 1 (MAPK pathway), Fig. 2 (PI3K pathway), and Fig. 3 (NF-κB pathway). While Fig. 4 shows bar chart for fold change of all statistically significant genes involved in more than one pathway. Negative and positive values denoted down-regulation and up-regulation of gene expression, respectively.

From the 43 genes identified, MNQ up-regulated 21 genes and down-regulated 22 genes. The identified 21 up-regulated genes were apoptotic genes (*BAD*, *EGR1*, *FOXO1*, *FOXO3*, *ITGB1*, *LTA*, *TNF*, *TNFRSF1A*, *TNFRSF10B*, *TRADD*, *YWHAH*), tumor suppressor genes (*APC*, *RB1*, and *TP53*) and cell cycle regulation genes (*CDKN1A*, *CDKN1B*, *CDKN2A*, *CDKN2D*, *MTOR*, *LAMTOR3*, and *PTEN*). The confirmed 22 down-regulated genes were anti-apoptotic genes (*HSPB1*, *NFKB1*, *NFKB2*, *NFKB1A*, *NFKB1E*, *RPS6KA1*), angiogenesis genes (*IL6*, *TIMP1*), cell cycle regulation genes (*ARAF*, *AKT2*, *AKT3*, *BRAF*, *EGFR*, *HRAS*, *IGF1*, *IGF1R*, *KRAS*, *NRAS*, *PDGFRA*), transcription factors (*FOS* and *JUN*), proto-oncogenes (*BCL3*).

Among all the 43 statistically significant expressed genes, 12 genes (*ARAF*, *BRAF*, *CDKN1A*, *CDKN1B*, *CDKN2A*, *CDKN2D*, *EGFR*, *KRAS*, *LAMTOR3*, *NRAS*, *RB1*, and *TP53*) are involved in MAPK pathway (Table 1), 14 genes (*AKT2*, *AKT3*, *APC*, *BAD*, *FOXO1*, *FOXO3*, *IGF1*, *IGF1R*, *ITGB1*, *MTOR*, *PDGFRA*, *PTEN*, *RPS6KA1*, and *YWHAH*) are involved in PI3K pathway (Table 2), 10 genes (*BCL3*, *IL6*, *LTA*, *NFKB2*, *NFKB1E*, *TIMP1*, *TNF*, *TNFRSF1A*, *TNFRSF10B*, and *TRADD*) are involved in NF-κB pathway (Table 3), and seven genes (*EGR1*, *FOS*, *HRAS*, *HSPB1*, *JUN*, *NFKB1*, and *NFKB1A*) are involved in all three pathways (Table 4).

Overall, the functions of 21 up-regulated and 22 down-regulated genes regulated by MNQ are listed in Tables 5 and 6, respectively.

**Fig. 5.**
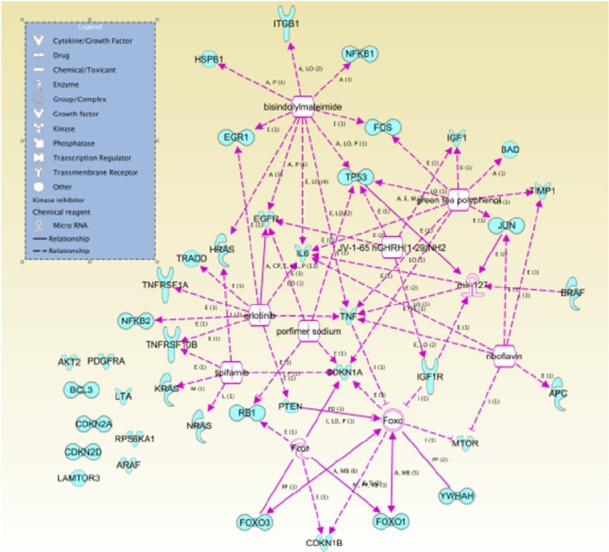
Network contains differentially expressed 43 genes. Top functions of the genes were related to molecular and cellular functions and physiological system development and function. Ingenuity Pathway Analysis network legend is on the left side.

The IPA software system enables systemic analysis of 43 statistically significant genes in a biologic context. Network of up/down-regulated genes were then algorithmically generated based on their inter-relationships. Five major networks were identified and included with functions related to cell death and survival, cellular development, cell cycle, proliferation and cell to cell signaling (Table 7). Canonical pathways were then identified and analyzed from the IPA libraries that were most significant to our common gene data set. Top statistically significant canonical pathways included PTEN, glioblastoma multiforme, PI3K/AKT, glioma signaling 1 and molecular mechanisms of cancer signaling pathways are shown in Table 8 (p < 0.05) and network contains differentially expressed 43 genes are shown in Fig. 5.

## Discussion

The common cancer pathways are MAPK, PI3K, NF-κB, STAT, NOTCH, TP53, RB1, WNT, and Hedgehog pathways. These pathways play crucial roles in survival and maintain tumourigenesis properties of cancer cells [21]. All these pathways are complement to each other forming a very complicated network, which has not been fully studied yet. In term of inhibiting signal transduction, suppression of multiple signaling pathways is better than suppression on single pathway at a time [11]. In a feedback loop theory, suppression of one signaling pathway would activate another pathway automatically. This is a phenomenon where cancer cells can prolong the survival as mutated cell [12]. Thus, this study focuses to determine the modulatory effects of MNQ on the key genes involved in MAPK, PI3K, and NF-κB pathways.

Overall, MNQ significantly regulated 43 genes (19.72%) from the total 218 cancer key genes studied in these three signaling pathways (Figs. 1‒3). These 43 genes that consist of 21 up-regulated genes (Table 5) and 22 down-regulated genes (Table 6) have different functions in regulating tumorigenesis in cancer cells (Table 7). The regulatory effects of these 43 genes in various cellular processes are discussed in following sections.

In this study, MNQ activated the apoptosis, tumor suppressor and cell cycle regulatory activities in MAPK pathway, where the genes of *LAMTOR3*, *TP53*, *RB1*, *CDKN1A*, *CDKN1B*, *CDKN2A* and *CDKN2D* and were up-regulated across all time points (Table 1, Fig. 1). MNQ induced the expression of P53 and RB1 transcription factors to exert its suppressive effect in preventing the progression of cell cycle in Raji cells. Upon the treatment of MNQ, MNQ could possibly cause DNA damage [22] and thus induce the expression of *P53*. In respond to DNA damage, P53 induces cyclin-dependent kinase inhibitors such as *CDKN1A*, *CDKN1B*, *CDKN2A*, *CDKN2D* that responsible for cell cycle arrest. The expression of late endosomal/lysosomal adaptor, MAPK and MTOR activator 3 (*LAMTOR3*) in MNQ-treated Raji could possibly explain that Raji cells tried to sustain their survival in the presence of the abovementioned genes in inducing cell cycle arrest and apoptosis. This phenomenon can be observed from Fig. 1 where the *LAMTOR3* expression were gradually increased in amount from 6 to 48 h treatment. In addition, *EGRF*, epidermal growth factor receptor involved in cell cycle was down-regulated by MNQ. Furthermore, proto-oncogene genes that are involved in cell growth and differentiation such as *ARAF*, *BRAF*, *KRAS*, *HRAS* and *NRAS* were also down-regulated across all time points. Overall, MNQ mainly caused cell cycle arrest, supressed cell proliferation and induced apoptosis in Raji cells via MAPK pathway.

In this study, MNQ activated tumor suppressor, apoptosis and cell cycle regulation through upregulating *PTEN*, *FOXO1*, *FOXO3*, *YWHAH*, *MTOR*, *ITGB1*, *BAD*, and *APC* genes in PI3K pathway (Table 2, Fig. 2), respectively. Among these up-regulated genes, adenomatous polyposis coli (*APC*) was found to be the most highest expressed gene across all time points. *APC* is a tumor suppressor that indirectly regulate a number of key genes involved in cell proliferation. Besides, it also plays a role in cell migration [23]. *PTEN* is another tumour suppressor gene up-regulated by MNQ that generally involved in the regulation of cell cycle, and it is known to be the target of many anticancer drugs. *FOXO1* and *FOXO3* have been reported to trigger apoptosis via regulating genes that responsible for cell death such as *BIM* and *PUMA* [24]. *BAD* is a known pro-apoptotic gene that initiate apoptosis. *YWHAH* encodes for 14-3-3 protein eta has been reported to regulate a wide variety of signaling pathways, and mainly mediated these signaling pathways by binding to phosphoserine-containing proteins. *MTOR* that encode for mTOR complex 1 and 2 proteins has been reported to regulate cell growth, cell proliferation, cell motility and cell survival [25]. *ITGB1* gene encodes Integrin beta-1 surface receptor that are involved in cell proliferation, cell adhesion and recognition as well as metastatic diffusion of tumor cells [26]. In our previous study [27], MNQ was demonstrated to induce apoptosis in A549 lung adenocarcinoma cell through oxidation triggered JNK and p38 MAPK signaling pathways. In this study, the abovementioned listed gene were up-regulated throughout all time points of treatment, this could explain Raji cells strived to survive upon the treatment of MNQ that creating the oxidative stress environment. Raji cells strived to survive via the abovementioned genes that are mainly involved in cell growth, proliferation, and survival. On the contrary, MNQ down-regulated *PDGFRA*, *AKT2*, *AKT3*, *RPS6KA1*, *IGF1*, and *IGF1R*, genes. *PDGFRA* gene has been reported to instruct the making of platelet-derived growth factor receptor alpha (PDGFRA). PDGFRA proteins activate signaling pathways to control many important cellular processes such as cell growth, division and survival. Mutation on *PDGFRA* has been reported in gastrointestinal stromal tumors and in chronic myeloid leukemia. Besides, highly phosphorylated *PDGFRA* has been observed in non‒small cell lung cancer and rhabdomyosarcoma [22], *AKT2* and *AKT3* are the serine/threonine kinases that regulate cellular metabolism, cell proliferation, cell survival, cell growth and angiogenesis. These genes are highly activated in many cancer cells. The AKT protein kinase has been indicated to transduce growth factors to oncogenes and target the proteins to induce tumor development. Thus, inhibition of the AKT kinase is workable in cancer prevention as down-regulation of AKT signaling is a strategy to prevent cancer formation [28]. Akt kinases has been known to inhibit FOXOs transcriptional functions and contribute to cell survival, cell growth and cell proliferation. *RPS6KA1* encodes ribosomal protein S6 kinase alpha-1 kinase that phosphorylate various substrates of the MAPK singling pathway, which is implicated in controlling cell growth and differentiation. *RPS6KA1* is also known as *RSK1* that present in cytoplasm. *RSK1* is an ERK effector that is involved mainly in nuclear signaling, cell growth, survival, and cell proliferation. It has been reported to regulate SRF, c-Fos, and Nur77 transcription factors. *RSK1* stimulated cell growth by regulating c-Fos and promotes cyclin D1 expression. Besides, RSK1 has been demonstrated to inhibit neuronal NO synthase in response to mitogenic signaling. *RSK1* is highly expressed in prostate and breast cancers. *RSK1* triggered survival signals via the Ras/ERK signaling pathway and protected cancer cells from apoptosis. *RSK1* is also involved in cell cycle regulation by phosphorylating CDKN1B and preventing CDKN1B translocation into nucleus and thus inhibited G1 progression. *RSK1* also promoted cell survival by suppressing the functions of pro-apoptotic proteins *BAD* and DAPK1. MNQ down-regulated insulin-like growth factor 1 (*IGF1*) and insulin-like growth factor 1 receptor (*IGF1R*) significantly. IGF family is a humoral mediator of growth hormone (GH) [29]. The IGF signaling pathway that control endocrine system and regulate cell growth and development has been reported to have a pathogenic role in cancer [30]. IGF has been reported to increase cancer cells growth and cells resistance to chemotherapy and radiation by decreasing functions of targeted agents in GH independent manner [31]. IGF1R is highly expressed and biologically active in small cell lung cancer, pediatric high-grade gliomas, and Ewing’s Sarcoma [32].

For NF-κB pathway, MNQ was found to up-regulate *TRADD*, *LTA*, *TNFRSF1A*, *TNFRSF10B*, and *TNF* genes, and down-regulate *NFKB2*, *NFKB1E*, *IL6*, *TIMP1*, and *BCL3* genes. LTA is a cytokine produced by lymphocytes that belong to tumor necrosis factor family. LTA bound to TNFRSF1A/TNFR1, TNFRSF1B/TNFBR, TNFRSF14 and induced apoptosis. TNFRSF1A is expressed in transformed cells to trigger apoptosis or inflammation [33]. Mutations in *TNFRSF1A* has been studied to display resistance towards apoptosis [34]. *TNFRSF1A* signals apoptosis through caspase-8 [35]. Tumor necrosis factor-α (*TNF*α) has been indicated to promote apoptosis in human endothelial cells through TNFRSF1A and trigger caspase-2 and p53 activation. It took part in variety cellular responses, such as survival, differentiation, proliferation, apoptosis, and migration [33]. TNFα is a pro-inflammatory cytokine regulated by TNFRSF1A and TNFRSF1B [36]. *TNFRSF1A* has been shown to promote the recruitment of TNFR-associated factors (TRAFs), FAS-associated via death domains (FADDs), and TNFR-associated via death domains (TRADDs). TNFRs interact with TRAFs, FADDs and TRADDS to control apoptosis. TNFα has been reported to bind with TNFRSF1A to induce apoptosis by activating caspase-8 [37]. Tumor necrosis factor receptor superfamily, member 10b (TNFRSF10B) is a member of the TNF-receptor superfamily, it has the similar function with TNFRSF1A. TNFRSF10B has been shown to interact with FADD [38], caspase 10, and caspase 8 and induced apoptosis in cancer cells. TRADD contains a death domain that interact with TNFRSF1A or TNFR1 to activate apoptosis. It has been reported to bind with TRAF2 to reduce the inhibitor-of-apoptosis proteins expression, and thus suppressed TRAF2-mediated apoptosis. TRADD activated apoptosis through FADD and caspase-8 activation [39]. STAT1, FADD, TNFRSF1A, and TNFRSF25 are the proteins interact with *TRADD*. *NFKB1*, *NFKB2*, *NFKB1A*, and *NFKB1E* are the genes encode for protein members of NF-κB family. Two angiogenesis genes of *IL6* and *TIMP1* were down-regulated by MNQ. IL6 is an inflammatory cytokine that modulate growth and differentiation in tumor cells through STAT3 signaling pathway. Overexpression of IL6 has been reported to be associated with tumor progression through inhibition of cancer cell apoptosis, stimulation of angiogenesis, and drug resistance. Clinical studies have been reported IL6 protein as a regulator that associate with many types of cancers such as multiple myeloma, non‒small cell lung adenocarcinoma, prostate cancer, colorectal cancer, renal cell carcinoma, breast cancer, and ovarian cancer [40,41]. Tissue inhibitor of metalloproteinases 1 (TIMP1) has become a marker of prognosis and indicator for checking the clinical response in cancer treatment because TIMP1 is elevated in breast, colon, and prostate cancer patient plasma [42,43]. *BCL3* was the only down-regulated proto-oncogene by MNQ. *BCL3* is a proto-oncogene deregulated in solid tumors. BCL3 has been revealed to induce proliferation and inhibit apoptosis. BCL3 activated STAT3, an aggressive oncogene in human cancer and promoted metastasis [44,45]. In leukemia cells, BCL3 has been demonstrated to up-regulate myc genes and lead to the formation of aggressive B-cell leukemia. Other report mentioned that NF-κB activated BCL3 to act as transcriptional co-activator. BCL3 contains two transactivating domain and can form homodimers with NF-κB1 (p50) or NF-κB2 (p52). It has been reported that BCL3 was up-regulated by cytokines such as TNFα, interleukin (IL)-1, IL-4, IL-6, IL-10, IL-12, and adiponectin [46-54]. These cytokines were activated by AP1 [46] and STAT3 [45,55]. In addition, BCL3 was down-regulated by p53 [56].

In a nutshell, MNQ up-regulated apoptotic genes (*BAD*, *EGR1*, *FOXO1*, *FOXO3*, *ITGB1*, *LTA*, *TNF*, *TNFRSF1A*, *TNFRSF10B*, *TRADD*, and *YWHAH*), tumor suppressor genes (*APC*, *RB1*, and *TP53*), and cell cycle genes (*CDKN1A*, *CDKN1B*, *CDKN2A*, *CDKN2D*, *MTOR*, *LAMTOR3*, and *PTEN*). On the contrary, MNQ down-regulated anti-apoptotic genes (*HSPB1*, *NFKB1*, *NFKB2*, *NFKB1A*, *NFKB1E*, and *RPS6KA1*), angiogenesis genes (*IL6* and *TIMP1*), cell cycle genes (*ARAF*, *AKT2*, *AKT3*, *BRAF*, *EGFR*, *HRAS*, *IGF1*, *IGF1R*, *KRAS*, *NRAS*, and *PDGFRA*), transcription factors (*FOS* and *JUN*), and proto-oncogenes (*BCL3*).

The findings of this study have revealed the capability of MNQ in regulating the key cancer genes in MAPK, PI3K, and NF-κB signaling pathways. The regulatory effects exerted by MNQ are targeting at cell proliferation, cell differentiation, cell transformation, induce apoptosis, reduce inflammatory responses, inhibits angiogenesis and metastasis. These results underline the need to further investigate the mechanism of actions of MNQ at each molecular level of these genes.

## Figures and Tables

**Table 1.** Up-regulated and down-regulated genes in MAPK pathway

Gene	Name	Time interval (h)			
		6	12	24	48
*BRAF*	V-raf murine sarcoma viral oncogene homolog B1	‒12.57	‒10.37	‒11.38	‒12.33
*KRAS*	V-Ki-ras2 Kirsten rat sarcoma viral oncogene homolog	‒10.35	‒11.34	‒11.02	‒10.47
*ARAF*	V-raf murine sarcoma 3611 viral oncogene homolog	‒5.37	‒6.43	‒7.45	‒4.27
*EGFR*	Epidermal growth factor receptor	‒5.32	‒6.52	‒12.37	‒10.69
*NRAS*	Neuroblastoma RAS viral (v-ras) oncogene homolog	‒4.17	‒6.53	‒5.60	‒5.53
*LAMTOR3*	Late endosomal/lysosomal adaptor, MAPK and MTOR activator 3	4.12	6.42	7.35	11.35
*TP53*	Tumor protein p53	5.29	6.35	6.23	4.35
*RB1*	Retinoblastoma 1	5.35	8.35	7.26	5.37
*CDKN1B*	Cyclin-dependent kinase inhibitor 1B (p27, Kip1)	5.44	5.29	6.15	6.31
*CDKN2D*	Cyclin-dependent kinase inhibitor 2D (p19, inhibits CDK4)	5.95	6.87	5.64	6.35
*CDKN1A*	Cyclin-dependent kinase inhibitor 1A (p21, Cip1)	7.47	8.32	10.45	7.37
*CDKN2A*	Cyclin-dependent kinase inhibitor 2A (melanoma, p16, inhibits CDK4)	10.11	9.75	8.26	5.38

MAPK, mitogen-activated protein kinase.

**Table 2.** Up-regulated and down-regulated genes in PI3K pathway

Gene	Name	Time interval (h)			
		6	12	24	48
*PDGFRA*	Platelet-derived growth factor receptor, alpha polypeptide	‒10.35	‒8.65	‒9.86	‒10.27
*AKT2*	V-akt murine thymoma viral oncogene homolog 2	‒7.31	‒9.35	‒20.21	‒12.37
*AKT3*	V-akt murine thymoma viral oncogene homolog 3 (protein kinase B, gamma)	‒6.97	‒9.34	‒12.93	‒5.32
*RPS6KA1*	Ribosomal protein S6 kinase, 90 kDa, polypeptide 1	‒5.97	‒6.32	‒4.29	‒6.30
*IGF1*	Insulin-like growth factor 1 (somatomedin C)	‒5.90	‒6.28	‒7.11	‒6.34
*IGF1R*	Insulin-like growth factor 1 receptor	‒5.82	‒6.28	‒7.34	‒6.93
*PTEN*	Phosphatase and tensin homolog	4.36	7.98	10.33	9.36
*FOXO1*	Forkhead box O1	4.4	5.28	5.39	5.28
*FOXO3*	Forkhead box O3	4.88	5.20	10.37	8.90
*YWHAH*	Tyrosine 3-monooxygenase/tryptophan 5-monooxygenase activation protein, eta polypeptide	5.10	6.33	7.92	8.44
*MTOR*	Mechanistic target of rapamycin (serine/threonine kinase)	6.13	6.32	7.94	5.24
*ITGB1*	Integrin, beta 1 (fibronectin receptor, beta polypeptide, antigen CD29 includes MDF2, MSK12)	6.38	5.38	5.19	6.32
*BAD*	BCL2-associated agonist of cell death	7.69	6.34	5.39	8.36
*APC*	Adenomatous polyposis coli	12.81	18.4	12.45	11.45

PI3K, phosphoinositide 3-kinase.

**Table 3.** Up-regulated and down-regulated genes in NF-κB pathway

Gene	Name	Time interval (h)			
		6	12	24	48
*NFKB2*	Nuclear factor of kappa light polypeptide gene enhancer in B-cells 2 (p49/p100)	‒5.38	‒17.35	‒12.85	‒10.43
*NFKB1E*	Nuclear factor of kappa light polypeptide gene enhancer in B-cells inhibitor, epsilon	‒4.88	‒5.55	‒6.23	‒9.62
*IL6*	Interleukin 6 (interferon, beta 2)	‒4.85	‒4.58	‒4.59	‒5.04
*TIMP1*	TIMP metallopeptidase inhibitor 1	‒4.33	‒4.03	‒3.97	‒4.05
*BCL3*	B-cell CLL/lymphoma 3	‒4.29	‒4.97	‒5.38	‒5.39
*TRADD*	TNFRSF1A-associated via death domain	4.03	5.97	5.39	4.09
*LTA*	Lymphotoxin alpha (TNF superfamily, member 1)	6.32	7.39	4.87	5.39
*TNFRSF1A*	Tumor necrosis factor receptor superfamily, member 1A	6.78	6.54	9.22	7.48
*TNFRSF10B*	Tumor necrosis factor receptor superfamily, member 10b	7.29	6.49	7.37	6.09
*TNF*	Tumor necrosis factor	8.26	10.84	11.46	12.59

NF-κB, nuclear factor кB.

**Table 4.** Down-regulated genes in MAPK, PI3K, or NF-κB pathways

Gene	Name	Pathway involved	Time interval (h)			
			6	12	24	48
*HSPB1*	Heat shock 27 kDa protein 1	MAPK, PI3K	‒6.96	‒6.30	‒7.22	‒6.39
*JUN*	JUN proto-oncogene	MAPK, PI3K, NF-κB	‒5.02	‒5.30	‒5.37	‒6.02
*NFKB1*	Nuclear factor of kappa light polypeptide gene enhancer in B-cells 1	PI3K, NF-κB	‒4.89	‒5.93	‒4.95	‒5.94
*FOS*	FBJ murine osteosarcoma viral oncogene homolog	MAPK, PI3K, NF-κB	‒4.28	‒4.39	‒5.29	‒6.47
*HRAS*	V-Ha-ras Harvey rat sarcoma viral oncogene homolog	MAPK, PI3K, NF-κB	‒4.28	‒4.30	‒5.38	‒6.34
*NFKB1A*	Nuclear factor of kappa light polypeptide gene enhancer in B-cells inhibitor, alpha	PI3K, NF-κB	‒4.26	‒3.95	‒4.39	‒4.88
*EGR1*	Early growth response 1	MAPK,NF-κB	5.38	4.48	8.32	5.32

MAPK, mitogen-activated protein kinase; PI3K, phosphoinositide 3-kinase; NF-κB, nuclear factor кB.

**Table 5.** Functions of 21 up-regulated genes by MNQ

Gene	Description	Pathway	Function
*TP53*	Tumor protein p53	MAPK	Tumor suppressor
*RB1*	Retinoblastoma 1	MAPK	Tumor suppressor
*CDKN1B*	Cyclin-dependent kinase inhibitor 1B (p27, Kip1)	MAPK	Suppress tumor growth and prevent cell cycle progression
*CDKN1A*	Cyclin-dependent kinase inhibitor 1A (p21, Cip1)	MAPK	Suppress tumor growth and prevent cell cycle progression
*CDKN2D*	Cyclin-dependent kinase inhibitor 2D (p19, inhibits CDK4)	MAPK	Important tumor suppressor gene in cell cycle regulation
*CDKN2A*	Cyclin-dependent kinase inhibitor 2A (melanoma, p16, inhibits CDK4)	MAPK	Important tumor suppressor gene in cell cycle regulation
*LAMTOR3*	Late endosomal/lysosomal adaptor, MAPK and MTOR activator 3	MAPK	Activation of MAPK2 and MTOR Induce cell proliferation
*TRADD*	TNFRSF1A-associated via death domain	NF-κB	Induction of apoptosis, suppresses TRAF2 mediated apoptosis
*LTA*	Lymphotoxin alpha (TNF superfamily, member 1)	NF-κB	Induce apoptosis in gastric cancer
*TNFRSF1A*	Tumor necrosis factor receptor superfamily, member 1A	NF-κB	Death domain receptor, transducer for apoptosis signal
*TNFRSF10B*	Tumor necrosis factor receptor superfamily, member 10b	NF-κB	Death domain receptor, transducer for apoptosis signal
*TNF*	Tumor necrosis factor	NF-κB	Death domain receptors, induce apoptosis, activates MAPK and NF-κB pathway
*APC*	Adenomatous polyposis coli	PI3K	Tumor suppressor mutated in colon cancer
*BAD*	BCL2-associated agonist of cell death	PI3K	Induce apoptosis in breast cancer
*FOXO1*	Forkhead box O1	PI3K	Tumor suppressors in a variety of cancers, transcriptional activator that regulates apoptosis and cell cycle progression, activating BCL2
*FOXO3*	Forkhead box O3	PI3K	Tumor suppressors in a variety of cancers, transcriptional activator that triggers apoptosis in the absence of survival factors
*ITGB1*	Integrin, beta 1 (fibronectin receptor, beta polypeptide, antigen CD29 includes MDF2, MSK12)	PI3K	Involve in tumor progression and metastasis
*MTOR*	Mechanistic target of rapamycin	PI3K	Cell cycle arrest and immune-suppressive effects
*YWHAH*	Tyrosine 3-monooxygenase/tryptophan 5-monooxygenase activation protein, eta polypeptide	PI3K	Relates to tumor cell proliferation and malignant outcome of gastric carcinoma
*PTEN*	Phosphatase and tensin homolog	PI3K	Tumor suppressor gene, regulating cell cycle
*EGR1*	Early growth response 1	MAPK, NF-κB	Transcription factor, suppress cell growth, transformation, and induce apoptosis

MNQ, 2-methoxy-1,4-naphthoquinone; MAPK, mitogen-activated protein kinase; NF-κB, nuclear factor кB; PI3K, phosphoinositide 3-kinase.

**Table 6.** Functions of 22 down-regulated genes by MNQ

Gene	Description	Pathway	Function
*BRAF*	V-raf murine sarcoma viral oncogene homolog B1	MAPK	Proto-oncogene involved in cell growth and differentiation of melanoma
*NRAS*	Neuroblastoma RAS viral (v-ras) oncogene homolog	MAPK	Proto-oncogene regulating cell division in response to growth factor stimulation
*ARAF*	V-raf murine sarcoma 3611 viral oncogene homolog	MAPK	Proto-oncogene involved in cell growth and development
*KRAS*	V-Ki-ras2 Kirsten rat sarcoma viral oncogene homolog	MAPK	Proto-oncogene regulating cell division in response to growth factor stimulation
*EGFR*	Epidermal growth factor receptor	MAPK	Cell cycle regulation
*NFKB2*	Nuclear factor of kappa light polypeptide gene enhancer in B-cells 2 (p49/p100)	NF-κB	Anti-apoptosis
*NFKB1E*	Nuclear factor of kappa light polypeptide gene enhancer in B-cells inhibitor, epsilon	NF-κB	Inhibitor in colon cancer
*TIMP1*	TIMP metallopeptidase inhibitor 1	NF-κB	Promote cell proliferation, angiogenesis, and anti-apoptotic
*IL6*	Interleukin 6 (interferon, beta 2)	NF-κB	Inflammatory cytokine, inhibit apoptosis, induce angiogenesis
*BCL3*	B-cell CLL/lymphoma 3	NF-κB	Proto-oncogenes, activates by NF-κB
*AKT2*	V-akt murine thymoma viral oncogene homolog 2	PI3K	Cell proliferation, anti-apoptosis
*AKT3*	V-akt murine thymoma viral oncogene homolog 3 (protein kinase B, gamma)	PI3K	Cell proliferation, anti-apoptosis
*RPS6KA1*	Ribosomal protein S6 kinase, 90kDa, polypeptide 1	PI3K	Interact with NFkB1A and MAPK1, important mediator of survival signals that protect cells from undergoing apoptosis
*IGF1*	Insulin-like growth factor 1 (somatomedin C)	PI3K	Cell cycle regulation
*IGF1R*	Insulin-like growth factor 1 receptor	PI3K	Cell cycle regulation
*PDGFRA*	Platelet-derived growth factor receptor, alpha polypeptide	PI3K	Cell cycle regulation
*FOS*	FBJ murine osteosarcoma viral oncogene homolog	MAPK, PI3K, NF-κB	Transcription factors
*HRAS*	V-Ha-ras Harvey rat sarcoma viral oncogene homolog	MAPK, PI3K, NF-κB	Proto-oncogene regulating cell division in response to growth factor stimulation
*JUN*	JUN proto-oncogene	MAPK, PI3K, NF-κB	Transcription factors
*NFKB1*	Nuclear factor of kappa light polypeptide gene enhancer in B-cells 1	PI3K, NF-κB	Anti-apoptosis
*NFKB1A*	Nuclear factor of kappa light polypeptide gene enhancer in B-cells inhibitor, alpha	PI3K, NF-κB	Rel protein inhibitor in colon cancer
*HSPB1*	Heat shock 27 kDa protein 1	MAPK, PI3K	Inhibition of apoptosis, regulation of cell development, and cell differentiation, cytoprotection, and support of cell survival under stress conditions, enhances the activation of the NF-κB pathway

MNQ, 2-methoxy-1,4-naphthoquinone; MAPK, mitogen-activated protein kinase; NF-κB, nuclear factor кB; PI3K, phosphoinositide 3-kinase.

**Table 7.** Molecular and cellular functions

Name	p-value	No. of molecules
Cell death and survival	5.58E-14‒2.02E-38	40
Cellular development	3.95E-14‒2.60E-37	40
Cell cycle	5.33E-14‒1.66E-33	35
Cellular growth and proliferation	3.95E-14‒4.31E-33	39
Cell-to-cell signaling and interaction	1.12E-14‒1.81E-29	32

**Table 8.** List of the genes in most significantly up/down-regulated top five canonical pathways

Pathway	‒Log (p-value)	Ratio	Overlap, n (%)	Molecules
PTEN signaling	2.77E01	1.44E-01	17/118 (14.4)	*ITGB1, AKT2, NRAS, YWHAH, BAD, HRAS, KRAS, NFKB2, NFKB1, PTEN, FOXO1, CDKN1A, FOXO3, PDGFRA, IGF1R, CDKN1B, EGFR*
		1.44 × 10^-1^		
Glioblastoma multi-forme signaling	2.6E01	1.16E-01	17/146 (11.6)	*TP53, CDKN2A, AKT2, NRAS, HRAS, KRAS, APC, PTEN, RB1, MTOR, IGF1, FOXO1, CDKN1A, PDGFRA, IGF1R, CDKN1B, EGFR*
PI3K/AKT signaling	2.52E01	1.3E-01	16/123 (13.0)	*TP53, ITGB1, AKT2, NRAS, YWHAH, BAD, HRAS, KRAS, NFKB2, NFKB1, PTEN, MTOR, FOXO1, FOXO3, CDKN1A, CDKN1B*
Glioma signaling molecular	2.5E01	1.58E-01	15/95 (15.8)	*TP53, CDKN2A, AKT2, NRAS, HRAS, KRAS, PTEN, RB1, MTOR, CDKN2D, IGF1, CDKN1A, PDGFRA, IGF1R, EGFR*
Mechanisms of cancer	2.42E01	5.48E-02	20/365 (5.5)	*TP53, ITGB1, CDKN2A, AKT2, NRAS, BAD, HRAS, KRAS, NFKB2, NFKB1, APC, BRAF, RB1, FOS, MTOR3, CDKN2D, JUN, FOXO1, CDKN1A, CDKN1B*

PI3K, phosphoinositide 3-kinase.
